# Risk of congenital anomalies in pregnant users of statin drugs

**DOI:** 10.1111/j.1365-2125.2007.02905.x

**Published:** 2007-05-15

**Authors:** Benjamin Ofori, Evelyne Rey, Anick Bérard

**Affiliations:** Research Center, CHU Sainte-Justine Montreal, Quebec, Canada; University of Montreal Montreal, Quebec, Canada

**Keywords:** antilipademic agents, congenital anomalies, pregnancy, pregnancy registry, statins

## Abstract

**What is already known about this subject**

- Cholesterol is known to be essential for fetal development.
- Statins, which inhibit cholesterol production, have therefore been considered as potential teratogens and are contraindicated in pregnancy.
- Data available thus far on the risks of congenital anomalies associated with statin therapy have come from non-analytic postmarketing surveillance studies.
- Given the increasing use of statins in women of childbearing age, there is a need for a population-based study on the risks of congenital anomalies associated with gestational statin use.

**What this study adds**

- In this pharmacoepidemiological study, we determined the risk of congenital anomalies in women who filled prescriptions for statins during the first trimester of pregnancy, compared with women who had stopped statins before pregnancy or those who used fibrates during pregnancy.
- We found no evidence of an increased risk of fetal anomalies among first-trimester statin users, or any discernable pattern of congenital anomalies among live births.
- However, in the absence of outcome data on nonlive births, conclusions remain uncertain.

**Aims:**

Evidence from animal studies suggests that statin medications should not be taken during pregnancy. Our aim was to examine the association between the use of statins in early pregnancy and the incidence of congenital anomalies.

**Methods:**

A population-based pregnancy registry was built. Three study groups were assembled: women prescribed statins in the first trimester (group A), fibrate/nicotinic acid in the first trimester (group B) and statins between 1 year and 1 month before conception, but not during pregnancy (group C). Among live-born infants, we selected as cases infants with any congenital anomaly diagnosed in the first year of life. Controls were defined as infants with no congenital anomalies. The rate of congenital anomalies in the respective groups was calculated. Adjusted odds ratios (OR) and 95% confidence intervals (CI) were also calculated.

**Results:**

Our study group consisted of 288 pregnant women. Among women with a live birth, the rate of congenital anomalies was 3/64 (4.69%; 95% CI 1.00, 13.69) in group A, 3/14 in group B (21.43%; 95% CI 4.41, 62.57) and 7/67 in group C (10.45%; 95% CI 4.19, 21.53). The adjusted OR for congenital anomalies in group A compared with group C was 0.36 (95% CI 0.06, 2.18).

**Conclusion:**

We did not detect a pattern in fetal congenital anomalies or evidence of an increased risk in the live-born infants of women filling prescriptions for statins in the first trimester of pregnancy. Conclusions, however, remain uncertain in the absence of data from nonlive births.

## Introduction

High blood cholesterol has been shown to be a risk factor for coronary heart disease (CHD) and cardiovascular death [1]. The rise in obesity, physical inactivity, cigarette smoking, high-carbohydrate diets [2, 3] and Type 2 diabetes [4] may be implicated in the increasing numbers of young people who are now considered for treatment with antilipaemic drugs. Furthermore, a strong genetic component to elevated cholesterol has also been identified, prompting the early screening of young adults who may be at risk [5]. Heterozygous familial hypercholesterolaemia (HeFH) affects as many as 1 in 500 people with high prevalence in certain subpopulations such as people of Quebecois extraction. The incidence of HeFH in Quebec is about 2.5-fold higher than in the rest of Canada [5].

Pregnant women or those likely to become pregnant clearly fall within the populations that might become targets for antilipaemic drug therapy. During normal pregnancy, there is a steady physiological increase in serum lipid concentrations, so that by the third trimester triglyceride levels are 300–400% higher and cholesterol levels 25–90% higher than in the nonpregnant state [6]. Nevertheless, the recommendation is for lipid-lowering agents (especially statins) to be discontinued before conception, or even during the 5–10 years while women have children because of the uncertainty over their safety for the fetus [3, 6]. This contraindication is not always respected and cases may arise where women are exposed to a statin during pregnancy, either by intent or inadvertently; indeed, 50% of pregnancies are unplanned [7] and many women may not be aware of their status until well into the critical weeks of organogenesis, by which time inadvertent exposure may already have occurred. Discontinuation of lipid-lowering medications for the relatively short duration of pregnancy is thought to have little impact on long-term therapy for primary hypercholesterolaemia [6] and is unlikely to affect outcomes of CHD. One exception may be in homozygous familial hypercholesterolaemia, where a case report has suggested an association between markedly elevated maternal serum cholesterol and fetal intrauterine growth restriction [8]. However, there is no evidence that given close obstetrical care, satisfactory outcomes for both mother and baby cannot be achieved [8–10].

No controlled studies have assessed the teratogenic potential of any statins in humans. However, case-series and postmarketing surveillance data are available. A recent notable report was on a case series [11] (corrigendum [12]) of all Food and Drug Administration (FDA) reports, literature and manufacturer data on statin exposures during pregnancy. Of 214 ascertained pregnancy exposures, of which 70 had evaluable outcomes, there were 22 cases of newborns with structural defects, 18 of which resulted in live births. A further analysis [13], which considered prospective reports of maternal exposure to simvastatin and lovastatin, was able to calculate the incidence rate of congenital anomalies. Among their reported live births, the incidence rate of congenital anomalies was 5/154 (3.2%).

Reports available to date have been based on voluntary submissions or from postmarketing surveillance monitoring; such studies are limited by their lack of appropriate comparator groups and under-reporting, and also usually lack denominator data, such as user population and drug exposure patterns, that would provide the exact number of patients exposed to the medicine and thus at risk of the adverse event [14]. Therefore, we undertook a study to examine the risk of congenital anomalies associated with gestational exposure to statins or other antilipaemic medications in a defined population of women in Quebec, Canada. Our objective was to study the relationship between three maternal exposure groups to antilipaemic medications, representing the most likely pattern of antilipaemic medication use in relation to pregnancy, and the outcome of any congenital anomalies in their live-born infants; these three groups consisted of those who stop statins before pregnancy, those who continue, and those who use nonstatin antilipaemic medications during the gestational period.

## Methods

### Data sources

**Figure 1:**
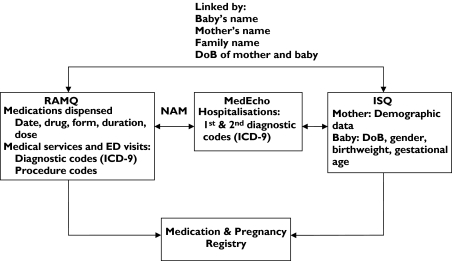
Linkage between administrative databases used in study. NAM, Numéro d'assurance maladie (unique personal identification number); ICD-9, International Classification of diseases, 9th revision; ED, emergency department; DoB, date of birth

We used three administrative databases of the Province of Quebec: the Régie de l'Assurance Maladie du Québec (RAMQ), Med-Echo, and the fichier des événements démographiques du Québec (birth and death registries) of l'Institut de la Statistique du Québec (ISQ) (Figure 1). The RAMQ database contains information on medical services (diagnoses and procedures) received by all Quebec residents. All diagnoses are classified according to the *International Classification of Diseases*, *9th revision* (ICD-9). The RAMQ covers all Quebec residents for the cost of physician visits, hospitalizations and procedures, but covers only a proportion of residents for the cost of prescribed medications. The RAMQ drug plan covers individuals ≥65 years old, recipients of social assistance (welfare beneficiaries) and workers and their families (adherents) who do not have access to a private insurance programme, accounting for approximately 43% of the overall Quebec population [15]. It is also estimated that 30% of women between 15 and 45 years of age in Quebec are covered by the RAMQ drug plan for their medication. The Med-Echo database is a provincial database which records hospitalization data for all Quebec residents; it also records gestational age for planned abortions, miscarriages and deliveries. The fichier des événements démographiques du Québec (ISQ) provides demographic variables on the mother, father and baby as well as birth weight and gestational age for live births and stillbirths. Linkage between the databases was achieved as illustrated in Figure 1. The RAMQ, Med-Echo and ISQ databases have previously been used for epidemiological research [16–19]. Data recorded in the medication database of the RAMQ have been evaluated and found to be comprehensive and valid [20]. The same has been found for medical diagnoses recorded in the Med-Echo database [21].

### Population

The RAMQ, Med-Echo and ISQ databases were linked together to create the ‘Medication and Pregnancy’ registry, which contains data on all pregnancies that occurred in Quebec between 1 January 1997 and 30 June 2003. This population-based pregnancy registry is composed of women with a diagnosis or procedure code related to pregnancy identified from data in the RAMQ or Med-Echo databases.

Within the ‘Medication and Pregnancy’ registry, women meeting the following eligibility criteria were included in the present study: they had to (i) be between 15 and 45 years of age at the date of entry in the registry defined as the first day of gestation, (ii) be continuously insured by the RAMQ drug plan for at least 12 months before the first day of gestation, and during their pregnancy, and (iii) have filled a prescription for a statin or fibrate or nicotinic acid in the year before or during pregnancy. If a woman had more than one pregnancy between 1997 and 2003, the first pregnancy meeting eligibility criteria was included.

### Definition of study groups

Within the study population, all women who had filled a prescription for a statin, a fibrate or nicotinic acid at any time from 1 year before the date of entry in the registry until the end of pregnancy were selected. The drugs considered were those reimbursed by the RAMQ at the time of the study, and included the following statins: atorvastatin, fluvastatin, lovastatin, pravastatin, rosuvastatin and simvastatin. The fibrates considered included fenofibrate, bezafibrate and gemfibrozil. In addition to these drugs, nicotinic acid was also considered.

Women were excluded if they had also filled a prescription for a category X drug, defined as a medication where studies in animals or humans have demonstrated fetal abnormalities or where there is evidence of fetal risk based on human experience or both, and the risk of the use of the drug in pregnant women clearly outweighs any possible benefit [22]. The category X drug group included: carbamazepine, phenytoin, valproic acid, lithium, acitretin, isotretinoin, antineoplastic agents (American Hospital Formulary System class 10 : 00.00), leflunomide and androgens (including danazol, testosterone and methyltestosterone).

Those pregnancies resulting in a live birth were selected for our analysis and were identified by searching the RAMQ/Med-Echo databases for ICD-9 codes and procedure codes related to childbirth and normal delivery, and by searching the ISQ database. For descriptive purposes, other pregnancies were identified as terminating in either a specified abortion (ICD-9 codes: 635.0–635.9, 636.0–636.9, 779.6, identified at any time up to 60 days after the end of pregnancy, to allow for delayed records) or other outcome, including unspecified abortions, miscarriages and stillbirth.

We defined three study groups of interest as follows. Group A was defined as women who had filled prescriptions for statins only, before and during the first trimester of pregnancy, but who did not use fibrates or nicotinic acid; group B as women who had filled prescriptions for fibrates or nicotinic acid only, before and during the first trimester, but who did not use statins; group C as women who had filled prescriptions for statins only in the period between 1 year before conception and 1 month before conception, and who did not have any filled prescriptions for any antilipaemic medication (statins, fibrates or nicotinic acid) in the period between 1 month before conception and the end of pregnancy. These groups were purposefully selected so that they all currently or previously had an indication for antilipaemic medication treatment and represented the treatment groups of clinical interest, those who stop their statins before pregnancy, those who continue their statins and those who use nonstatin antilipaemic medications.

### Covariates

Socio-demographic variables (from the RAMQ/ISQ database) as determined at the end of pregnancy were added, including maternal age (defined as a continuous variable and a categorical variable), marital status (living alone *vs.* cohabiting), number of years of education achieved as a continuous variable and categorically defined as: secondary education not completed (<11 years); secondary education completed (11 years); post secondary education (12–15 years); and university education (≥16 years). RAMQ insurance status (welfare beneficiary *vs.* adherent) and place of residence (urban *vs.* rural) were also included. Healthcare utilization variables (RAMQ/Med-Echo databases) were selected as markers of health status; hospitalization and emergency department (ED) visits (yes/no) and number of prescribers ≥2 (yes/no) were determined for the year before and during pregnancy; the number of medical visits ≥3 (yes/no) was determined for the year before pregnancy; a visit to an obstetrician or gynaecologist (ob/gyn) (yes/no) was determined for the period during the pregnancy. Prenatal visits, defined as being ≤7 or as a continuous variable, was also considered. Those women who had had a pregnancy within the previous year were also identified.

The presence/absence of the following chronic co-morbidities were determined using the RAMQ/Med-Echo databases: the diagnosis of hypothyroidism (ICD-9 244.0) or the filling of prescriptions for thyroid medications [American Hospital Formulary Service (AHFS) 68:36.04] at any time before or during pregnancy; the diagnosis of diabetes in the year before pregnancy (ICD-9 250.0–250.9, 271.4, 790.2) or gestational diabetes diagnosed at ≥26 weeks of pregnancy (ICD-9 648.0, 648.8), or the filling of prescriptions for medications for diabetes in the 12 months before and during pregnancy, including insulins and oral hypoglycemics (AHFS 68 : 20.08, 68 : 20.20, 68 : 20.92); the diagnosis of chronic hypertension in the year before and during pregnancy (defined by the presence of ICD-9 codes 401.0–405.9, 362.1, 416.0, 437.2, 796.2), or the filling of prescriptions for any antihypertensive drugs (AHFS class 24 : 08), or gestational hypertension defined as a diagnosis made at ≥20 weeks of pregnancy and identified with ICD-9 codes 642.0–642.9. Both diabetes and hypothyroidism are causes of secondary hyperlipidaemia [23] and, together with hypertension, represent clinically important co-morbidities frequently found in pregnant women.

We also considered the number of different medications prescribed (>2, yes/no) other than statins or fibrates/nicotinic acid during pregnancy. Where such prescribed medications were considered, we took into account those prescriptions filled before pregnancy but whose duration overlapped into the period of pregnancy. The data collected on the babies included birth weight and gender (ISQ database). As there is an increased risk of prematurity in fetuses with a congenital anomaly [24], we also reported the percentage of premature births (<37 weeks gestational age at birth).

### Cases and controls

We investigated the presence of major and minor congenital anomalies in live-born babies. Cases were defined as infants born with any congenital anomaly. These were identified by searching the RAMQ and Med-Echo databases for ICD-9 codes 740.0–759.9 among those women with live births. The index date for case definition was considered as the end of pregnancy. However, we also searched for records of congenital anomalies made within the first 12 months of each infant's life, to allow for delayed detections or registrations and enhance the detection of anomalies. Controls were defined as infants with no (neither major nor minor) congenital anomaly documented in the first 12 months of life.

### Statistical analysis

Pregnancy outcomes in the three study groups are described. Further descriptive statistics were performed only for those women with live births. Descriptive statistics were also used to describe the population by case status. The congenital anomalies detected, stratified by study group, are also listed.

The incidence of congenital anomalies for each study group was calculated using the number of reported anomalies in mother–baby-linked live births as the numerator and the total number of mother–baby-linked live births for each group as the denominator. Confidence intervals (CIs) were calculated using the method of Haenszel *et al.* [25] for Poisson-distributed variables. The independent effect of the study group status (using group B as the reference group) and of maternal or infant characteristics on congenital anomalies was estimated by univariate logistic regression. These analyses were repeated on a subset comparing group A with group C (reference group). For each variable, crude numbers for cases and controls are displayed. Two multivariate logistic regression models were performed to control simultaneously for potential confounders. The first model included the entire study cohort stratified by study group, using group B as the reference group. The second model included groups A and C, using group C as the reference group. Covariates for inclusion were selected by the backward stepwise method with a significance criterion for removal from the model of *P* > 0.25. Maternal age at end of pregnancy and the following measures of socioeconomic status were retained in the model: place of residence, insurance status, marital status and years of education achieved. To test for a curvilinear relationship between age and congenital anomaly, a quadratic term (age squared) was added to the linear term in a separate model. There were missing data in two variables, marital status (7.6%) and years of education achieved (8.3%). This was dealt with by assuming cohabitation for marital status and secondary education completed (11 years' education), where data were missing. We examined the effect on our model of reversing these assumptions and adopting living alone as the default for marital status, and for education assuming either secondary education not completed (≤10 years) or university education (≥16 years' education).

Descriptive and statistical analyses were performed using the SAS System for Windows V8.0 (SAS Institute Inc., Cary, NC, USA). Ethics approval was obtained from the Research Ethics Board of CHU Sainte-Justine and from the Commission for access to information of Quebec.

## Results

**Figure 2:**
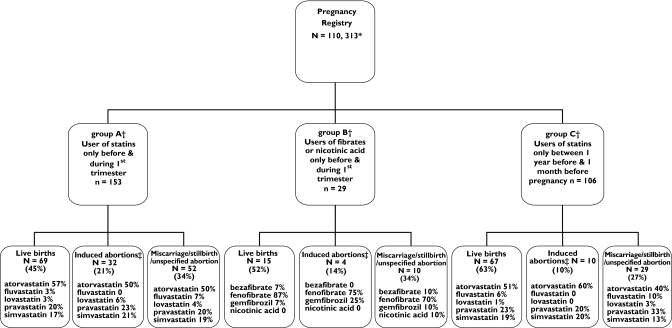
Pregnancy outcomes according to study group. *Women aged 15–45 years on first day of gestation; covered by Régie de l'Assurance Maladie du Québec drug insurance for ≥12 months before pregnancy and during pregnancy. †Women excluded; users of other known teratogens (category X drug; carbamazepine, phenytoin, valproic acid, lithium, acitretin, isotretinoin, antineoplastic agents, leflunomide and androgens). ‡Induced abortions identified by ICD-9 codes 635.0–635.9, 636.0–636.9, 779.6

The Medication and Pregnancy registry included 110 313 women. Of these, 153 women received a statin during the first trimester of pregnancy (group A), 29 received a fibrate or nicotinic acid in the first trimester of pregnancy (group B) and 106 women received a statin in the period between 1 year before conception and 1 month before conception (group C). The pregnancy outcomes for these three groups are described in Figure 2. The three most commonly filled prescriptions for a statin among the women in group A and group C were for atorvastatin, pravastatin and simvastatin. In all cases of first-trimester statin or fibrate use, prescriptions were not renewed in the second trimester.

We were able to perform a link of mother–baby data for 64 of the 69 known live births for group A. In group B mother–baby linkage was possible in 14 out of the 15 known live births, whereas in group C all mothers were successfully linked to babies (67/67). Further results refer to these groups.

The patterns of antilipaemic medications used are presented in Table 1. For group A, the patterns of statin use before pregnancy and during pregnancy were analysed separately. Five women made treatment changes: three in group A in the period before pregnancy and two in group C. In three cases, a switch was made to atorvastatin from pravastatin or fluvastatin, in one case a switch was made from simvastatin to atorvastatin then back to simvastatin, and in one case from fluvastatin to simvastatin. In Table 2, the frequency of the most commonly filled prescriptions in group A is compared with those in groups B and C.

**Table 2:** Comparison of commonly prescribed medications between group A and groups B and C

	**Group APrescribed statins only before and during first trimester, *n* = 64**	**Group BFibrates or nicotinic acid only prescribed before and during first trimester, *n* = 14**	**Group CStatins only, prescribed between 1 year before and 1 month before pregnancy, *n* = 67**
Antiemetics (%)	8.22	4.82	6.38
Blood glucose reagents (%)	7.31	8.43	6.91
Penicillins (%)	6.85	4.82	6.38
Antihypertensive medication (%)	4.57	3.61	2.13
Insulin (%)	4.11	7.23	3.72
Antifungals (%)	4.11	4.82	5.85
Nonsteroidal anti-inflammatory drugs (%)	3.65	1.2	7.45
Antidepressants (%)	3.65	2.41	2.13
Thyroid hormones (%)	3.65	1.20	1.60
Quinidine (%)	3.20	–	1.06

**Table 1:** Antilipaemic medications prescribed according to study group in women with live births

	Group APrescribed statins only before and during first trimester, *n* = 64*		Group BFibrates or nicotinic acid only prescribed before and during first trimester, *n* = 14*	Group CStatins only, prescribed between 1 year before and 1 month before pregnancy *n* = 67*†
	**Statins prescribed before pregnancy**	**Statins prescribed during pregnancy**		
*Cholesterol medication*				
Atorvastatin, no. (%)	32 (53.33)	33 (60.00)		36 (52.17)
Fluvastatin, no. (%)	3 (5.00)†	2 (3.64)		4 (5.80)¶
Lovastatin, no. (%)	2 (3.33)	2 (3.64)		1 (1.45)
Pravastatin, no. (%)	12 (20.00)§	11 (20.00)		15 (21.74)††
Simvastatin, no. (%)	11 (18.33)**	7 (12.73)		13 (18.84)
Bezafibrate, no. (%)			1 (7.14)	
Fenofibrate, no. (%)			12 (85.71)	
Gemfibrozil, no. (%)			1 (7.14)	

* Women with live births and complete mother–baby linkage data. Numbers of women are fewer than in *Figure 1*, as there were a few women with incomplete linkage of data with their babies.

† Includes one woman with a twin birth.

‡ One woman used fluvastatin and changed to simvastatin.

§ One woman used pravastatin, then changed to atorvastatin.

¶ One woman used fluvastatin, then changed to atorvastatin.

** One woman used simvastatin, then changed to atorvastatin, then back to simvastatin.

†† One woman used pravastatin, then changed to atorvastatin.

The rate of congenital anomalies in group A was 3/64 (4.69%; 95% CI 1.00, 13.69) (Table 3). The rate in group B was 3/14 (21.43%; 95% CI 4.41, 62.57) and in group C, 7/67 (10.45%; 95% CI 4.19, 21.53). By comparison, the live-birth congenital anomaly rate in the rest of our registry was 6.97% (95% CI 6.77, 7.17) [26]. Table 3 also shows variable information for the entire study cohort according to case and control status. Crude OR and 95% CI for the occurrence of any major or minor congenital anomaly are shown as an indication of the uncertainty around the estimation of risk. Similarly, Table 4 displays data for groups A and C.

**Table 4:** Characteristics of cases and controls in two study groups and risk of congenital anomalies in relation to characteristics

	**Group A *n* = 64**		**Group C (ref. group), *n* = 67**				
	**Cases, *n* (%)**	**Controls, *n* (%)**	**Cases, *n* (%)**	**Controls, *n* (%)**	**Crude OR**	**95% CI**	***P*-*value***
Risk of congenital anomalies in Group A *Maternal age at end of pregnancy, years*	3 (4.69)	61 (95.31)	7 (10.45)	60 (89.55)	0.42	0.10, 1.71	0.23
≤18	0 (0)	0 (0)	0 (0)	1 (1.67)	<0.001	–	0.99
19–25	2 (66.67)	6 (9.84)	1 (14.29)	11 (18.33)	4.18	0.77, 22.53	0.10
26–34	0 (0)	35 (57.38)	3 (42.86)	36 (60.00)	Reference		
≥35	1 (33.33)	20 (32.79)	3 (42.86)	12 (20.00)	2.96	0.62, 13.99	0.17
*Socioeconomic information (at end of pregnancy)*							
Urban dweller (y/n)	3 (100.00)	44 (72.13)	6 (85.71)	43 (71.67)	3.52	0.43, 28.83	0.24
Welfare beneficiary (y/n)	2 (66.67)	20 (32.79)	2 (28.57)	26 (43.33)	1.09	0.29, 4.06	0.90
Living alone (y/n)	2 (66.67)	8 (13.11)	2 (28.57)	17 (28.33)	2.56	0.67, 9.77	0.17
*Education*							
Secondary education incomplete (<11 years)	2 (66.67)	22 (36.07)	2 (28.57)	17 (28.33)	Reference		
Secondary education completed	0 (0)	14 (22.95)	3 (42.86)	10 (16.67)	1.22	0.25, 5.92	0.81
Post secondary education (12–15 years)	1 (33.33)	22 (36.07)	2 (28.57)	27 (45.00)	0.60	0.13, 2.83	0.51
University education	0 (0)	3 (4.92)	0 (0)	6 (10.00)	<0.001	–	0.97
*Maternal co-morbidities*							
Diabetes status* (y/n)	1 (33.33)	12 (19.67)	3 (42.86)	10 (16.67)	3.00	0.78, 11.54	0.11
Chronic or gestational hypertension or prescription for antihypertensives† (y/n)	0 (0)	15 (24.59)	3 (42.86)	8 (13.33)	1.83	0.44, 7.61	0.41
Hypothyroidism before or during pregnancy‡ (y/n)	1 (33.33)	7 (11.48)	1 (14.29)	4 (6.67)	2.50	0.47, 13.27	0.28
Different medications > 2 (excluding antilipaemic drugs) during pregnancy	2 (66.67)	31 (50.82)	2 (28.57)	25 (41.67)	0.77	0.21, 2.88	0.70
*Health services utilization*							
Prenatal visits ≤ 7	1 (33.33)	15 (24.59)	2 (28.57)	17 (28.33)	1.19	0.29, 4.89	0.81
Medical visits ≥ 3 in year prior to pregnancy	2 (66.67)	54 (88.52)	5 (71.43)	53 (88.33)	0.30	0.07, 1.32	0.11
Different prescribers ≥ 2 in year prior to and during pregnancy	3 (100.00)	52 (85.25)	7 (100.00)	51 (85.00)	–	–	0.96
ED visit or hospitalization in year prior to and during pregnancy (y/n)	3 (100.00)	48 (78.69)	4 (57.14)	50 (83.33)	0.55	0.13, 2.28	0.41
Visit to an OB/GYN during pregnancy (y/n)	3 (100.00)	51 (83.61)	6 (85.71)	48 (80.00)	2.00	0.24, 16.61	0.52
Pregnancy in previous year (y/n)	0 (0)	6 (9.84)	1 (14.29)	7 (11.67)	0.92	0.11, 7.88	0.94
Gestational age at delivery < 37 weeks	1 (33.33)	5 (8.20)	3 (42.86)	5 (8.33)	7.40	1.79, 30.65	0.01
Birth weight < 2500 g	1 (33.33)	7 (11.48)	1 (14.29)	5 (8.33)	2.271	0.43, 11.95	0.33
Gender of baby, male	1 (33.33)	33 (54.10)	3 (42.86)	32 (53.33)	0.574	0.15, 2.14	0.41

OR, odds ratio; 95% CI, 95% Confidence Interval. ED, Emergency department; OB/GYN, obstetrician and gynaecologist; y/n, yes/no.

* Diabetes status defined as a diagnosis of chronic diabetes in the year before pregnancy (ICD-9; 250.0–250.9, 271.4, 790.2) or gestational diabetes diagnosed at ≥26 weeks of pregnancy (ICD-9; 648.0, 648.8), or filled prescriptions for medications for diabetes in the 12 months before and during pregnancy.

† Chronic hypertension defined as diagnosis of hypertension in year before pregnancy (ICD-9; 401.0–405.9, 362.1, 416.0, 437.2, 796.2) or filled prescriptions for any antihypertensive drugs under the American Hospital Formulary Service (AHFS) class 24 : 08. Gestational hypertension defined as a diagnosis made at ≥20 weeks of pregnancy (ICD-9; 642.0–642.9).

‡ Hypothyroidism defined as ICD-9; 244.0 or the use of thyroid medication (AHFS 68 : 36.04) at any time before or during pregnancy.

**Table 3:** Risk of any congenital anomaly in relation to study group and characteristics

	**Cases,*n* (%)**	**Controls,*n* (%)**	**Crude OR**	**95% CI**	***P*-*value***
*Study group*					
Group B fibrates or nicotinic acid only prescribed before and during first trimester, *n* = 14*	3 (21.43)	11 (78.57)	Reference		
Group A statins only prescribed before and during first trimester, *n* = 64*	3 (4.69)	61 (95.31)	0.18	0.03, 1.01	0.05
Group C statins only prescribed between 1 year before and 1 month before pregnancy, *n* = 67*†	7 (10.45)	60 (89.55)	0.43	0.10, 1.91	0.27
*Maternal age at end of pregnancy, years*					
≤18	0 (0)	2 (1.52)	<0.001	–	0.99
19–25	4 (30.77)	18 (13.64)	5.63	1.16, 27.40	0.03
26–34	3 (23.08)	76 (57.58)	Reference		
≥35	6 (46.15)	36 (27.27)	4.22	1.00, 17.85	0.05
*Socioeconomic information (at end of pregnancy)*					
Urban dweller (y/n)	12 (92.31)	96 (72.73)	4.50	0.56, 35.86	0.15
Welfare beneficiary (y/n)	6 (46.15)	51 (38.64)	1.36	0.43, 4.28	0.60
Living alone (y/n)	6 (46.15)	28 (21.21)	3.18	0.99, 10.23	0.05
*Education*					
Secondary education incomplete (<11 years)	4 (30.77)	43 (32.58)	Reference		
Secondary education completed	5 (38.46)	27 (20.45)	1.99	0.49, 8.07	0.33
Post secondary education (12–15 years)	4 (30.77)	53 (40.15)	0.81	0.19, 3.43	0.78
University education	0 (0.00)	9 (6.82)	<0.001	–	0.98
*Maternal co-morbidities*					
Diabetes status‡ (y/n)	7 (53.85)	28 (21.21)	4.33	1.35, 13.93	0.01
Chronic or gestational hypertension or prescription for antihypertensives§ (y/n)	5 (38.46)	28 (21.21)	2.32	0.70, 7.65	0.17
Hypothyroidism before or during pregnancy¶ (y/n)	3 (23.08)	12 (9.09)	3.00	0.72, 12.41	0.13
Different medications > 2 (excluding antilipaemic drugs) during pregnancy	7 (53.85)	62 (46.97)	1.32	0.42, 4.13	0.64
*Health services utilization*					
Prenatal visits ≤7	5 (38.46)	36 (27.27)	1.67	0.51, 5.43	0.40
Medical visits ≤3 in year prior to pregnancy	10 (76.92)	115 (87.12)	0.49	0.12, 1.97	0.32
Different prescribers ≥2 in year prior to and during pregnancy	13 (100.00)	114 (86.36)	<0.001	–	0.97
ED visit or hospitalization in year prior to and during pregnancy (y/n)	9 (69.23)	107 (81.06)	0.53	0.15, 1.84	0.31
Visit to an OB/GYN during pregnancy (y/n)	12 (92.31)	107 (81.06)	2.80	0.35, 22.55	0.33
Pregnancy in previous year (y/n)	2 (15.38)	14 (10.61)	1.53	0.31, 7.63	0.60
Gestational age at delivery <37 weeks	6 (46.15)	11 (8.33)	9.43	2.69, 33.01	<0.001
Birth weight <2500 g	3 (23.08)	13 (9.85)	2.75	0.67, 11.27	0.16
Gender of baby, male	6 (46.15)	70 (53.03)	0.76	0.24, 2.38	0.64

OR, odds ratio; 95% CI, 95% Confidence Interval.

* Women with live births and complete mother–baby linkage data.

† Includes one woman with a twin birth. ED, Emergency department; OB/GYN, obstetrician and gynaecologist; y/n, yes/no.

‡ Diabetes status defined as a diagnosis of chronic diabetes in the year before pregnancy (ICD-9; 250.0–250.9, 271.4, 790.2) or gestational diabetes diagnosed at ≥26 weeks of pregnancy (ICD-9; 648.0, 648.8), or filled prescriptions for medications for diabetes in the 12 months before and during pregnancy.

§ Chronic hypertension defined as diagnosis of hypertension in year before pregnancy (ICD-9; 401.0–405.9, 362.1, 416.0, 437.2, 796.2) or filled prescriptions for any antihypertensive drugs under the American Hospital Formulary Service (AHFS) class 24 : 08. Gestational hypertension defined as a diagnosis made at ≥20 weeks of pregnancy (ICD-9; 642.0–642.9).

¶ Hypothyroidism defined as; ICD-9; 244.0 or the use of thyroid medication (AHFS 68:36.04) at any time before or during pregnancy.

The first multivariate analysis of the entire study cohort, stratified by study group (using group B as the reference group), included the variables; maternal age at end of pregnancy (as a continuous variable), all the socioeconomic variables as previously described, and the following categorical indicators of co-morbid diseases: diabetes, hypertension, hypothyroidism, comedications and medical visits before pregnancy and history of pregnancy in previous year. The adjusted OR for congenital anomalies for group A was 0.79 (95% CI 0.10, 6.02) and for group C 1.74 (95% CI 0.27, 11.27), when compared with group B. In the second multivariate analysis, which included groups A and C, using group C as the reference group, the final model was adjusted for age at end of pregnancy (as a continuous variable), socioeconomic variables as previously described, and the following categorical indicators of co-morbid diseases: diabetes, hypothyroidism, comedications, medical visits before pregnancy, low birth weight and baby's gender. The adjusted OR for groupA was 0.36 (95% CI 0.06, 2.18), when compared with group C.

There was no evidence of a nonlinear trend with the quadratic term for maternal age entered into the model (*P* for the quadratic term in the first multivariate analysis = 0.22; *P* for the quadratic term in second multivariate analysis = 0.50). Under the alternative assumptions for missing data, i.e. living alone by default and education ≤10 years, the point estimates for groups A and C in the first multivariate analysis of the entire study cohort stratified by study group were adjusted OR = 0.72 (0.10, 5.31) and adjusted OR = 1.51 (0.25, 9.26), respectively. If ≥16 years of education was assumed, then the adjusted OR estimates were 0.73 (95% CI 0.10, 5.48) and 1.54 (95% CI 0.24, 9.79) for groups A and C, respectively. The adjusted ORs in the second multivariate analysis (comparing group A with C) under these various assumptions remained unchanged.

The most common anomalies detected were related to anomalies of the heart (ICD-9 745, 746) (Table 5). In group A, the three anomalies detected were an unspecified anomaly of the heart, a ventricular septal defect and an atrial septal defect. The statin medications prescribed in these three cases were lovastatin, atorvastatin and simvastatin. Despite the prevalence in use of pravastatin (20%) during the first trimester, there were no congenital anomalies associated with this medication among the live births. A variety of unrelated anomalies were represented in group B: one cardiac anomaly, one musculoskeletal anomaly and one case of tuberous sclerosis, and an anomaly of the eye. Similarly, a variety of unrelated anomalies was represented in group C, including musculoskeletal, limb, cardiac and respiratory anomalies, suggesting no specific pattern in congenital anomalies. In all three groups, the dose of medications being taken was the standard recommended therapeutic dose [27].

**Table 5:** Description of congenital anomalies in study groups

	**Congenital anomalies by study group**	**Antilipaemic drug**	**Daily dose (mg)**	**Maternal age at end of pregnancy (years)**	Diabetes status*(y/n)	**Gestational age at delivery (weeks)**	**Gender of baby (f/m)**
ID	A. Statins only prescription, before and during first trimester, *n* = 3						
1	Unspecified anomaly of the heart (ICD-9; 746.9)	Lovastatin†	20	35	n	34	f
2	Ventricular septal defect (ICD-9; 745.4)	Simvastatin†	10	24	n	41	f
2	Unspecified defect of septal closure (ICD-9; 745.9)	Simvastatin†	10				
3	Other specified anomaly of heart (ICD-9; 746.8)	Atorvastatin†	10	21	y	37	m
3	Ostium secundum type atrial septal defect (ICD-9: 745.5)	Atorvastatin†	10				
ID	B. Fibrates or nicotinic acid only prescription, before and during first trimester, *n* = 3						
4	Transposition of great vessels (ICD-9; 745.1)	Fenofibrate†	200	41	y	36	m
4	Unspecified anomaly of heart (ICD-9; 746.9)	Fenofibrate†	200				
4	Congenital anomaly unspecified (ICD-9; 759.9)	Fenofibrate†	200				
5	Unspecified anomaly of musculoskeletal system (ICD-9; 756.9)	Fenofibrate†	200	37	y	40	m
6	Tuberous sclerosis (ICD-9; 759.5)	Fenofibrate†	200	20	y	34	f
6	Unspecified anomaly of eye (ICD-9; 743.9)	Fenofibrate†	200				
ID	C. Statins only prescription, between 1 year before and 1 month before pregnancy, *n* = 7						
7	Unspecified anomaly of musculoskeletal system (ICD-9: 756.9)	Simvastatin†	10	23	n	40	f
8	Unspecified anomaly of unspecified limb (ICD-9: 755.9)	Atorvastatin†	10	30	y	36	m
9	Ventricular septal defect (ICD-9: 745.4)	Atorvastatin†	10	37	y	38	f
10	Ostium secundum type atrial septal defect (ICD-9: 745.5)	Simvastatin†	20	37	y	30	m
11	Unspecified anomaly of respiratory system (ICD-9: 748.9)	Lovastatin†	20	27	n	36	m
12	Ventricular septal defect (ICD-9: 745.4)	Atorvastatin†	10	37	n	37	f
13	Varus deformities of feet (ICD-9: 754.5)	Atorvastatin‡	40	26	n	41	f

* Diabetes status defined as a diagnosis of chronic diabetes in the year before pregnancy (ICD-9; 250.0–250.9, 271.4, 790.2) or gestational diabetes diagnosed at ≥26 weeks of pregnancy (ICD-9; 648.0, 648.8), or the filling of prescriptions for medications for diabetes in the 12 months before and during pregnancy.

† Represent prescriptions for antilipaemic medications filled during the first trimester (some women may also have filled prescriptions before the first trimester but the duration of treatment overlapped into the first trimester).

‡ Represent prescriptions for antilipaemic medications lasting up to 1 month before pregnancy. Subjects in this group did not fill prescriptions for antilipaemic medications during pregnancy. Tuberous sclerosis: familial neurocutaneous disease characterized by epilepsy, mental deterioration, adenoma sebaceum, nodules and sclerotic patches on the cerebral cortex, retinal tumours, tumours of the heart or kidneys.

## Discussion

In this study, we found that the overall incidence of congenital anomalies in pregnancies where prescriptions for statins had been filled in the first trimester of pregnancy (3/64, 4.69%) was not statistically greater than the incidence in those pregnancies where prescriptions for fibrates only had been filled in the first trimester (3/14, 21.43%), or where the statins had been stopped at least 1 month before conception (7/67, 10.45%). There was no evident pattern in the types of congenital anomalies among the live births. However, the sample size of our study was small and lacked sufficient power to detect small increases in overall risk among those taking statins during pregnancy. Furthermore, the number of cases ascertained was also very small. We acknowledge that this is a limitation in drawing inferences from the statistical tests of significance. Nevertheless, this is an issue common to many such studies published so far.

The congenital anomalies detected in the three cases where statin prescriptions had been filled in the first trimester involved lovastatin, simvastatin and atorvastatin. No malformations were detected among the 11 infants exposed to pravastatin. One prevailing theory is that statins with high affinity for lipid environments (including, simvastatin > lovastatin > atorvastain > cerivastatin > fluvastatin) more readily enter extrahepatic tissues, including the embryo during pregnancy, and thus have a greater potential to downregulate cholesterol biosynthesis [11]. In case reports of statin-exposed pregnancies and outcomes [11], all cases of adverse outcomes at birth were associated with lipophilic statins; no malformations were reported among infants exposed to pravastatin.

Studies on humans to date have not shown statins to be potent teratogens or to cause particular patterns of congenital anomalies. In our study, the 3/64 cases of live babies with congenital anomalies associated with filling prescriptions for statins in the first trimester of pregnancy consisted of an unspecified anomaly of the heart, a ventricular septal defect and an atrial septal defect. Similarly, another study [11] involving the evaluation of reports on first-trimester statin exposures from the FDA's Medical Products Reporting Program reported 18 live births with structural anomalies. Detected anomalies included: limb deficiencies (*n* = 2), cleft palate (*n* = 1), cleft lip (*n* = 2), oesophageal atresia (*n* = 1), spina bifida (*n* = 1), duodenal atresia (*n* = 1), polydactyly (*n* = 1), hypospadias (*n* = 1), constriction of pyelourethral junction (*n* = 1), clubbed foot (1), vertebral, anal, tracheo-oesophageal, renal (VATER) (*n* = 1), aqueductal stenosis (*n* = 1), ‘severe deformity’ (*n* = 1), microtia (*n* = 1), ‘cardiovascular defect’ (*n* = 1) and an atrial/ventricular septal defect/aortic hypoplasia (as subsequently corrected [12]). The statins implicated were atorvastatin, simvastatin and lovastatin.

Most studies of associations of human birth defects, such as ours, are undertaken in live births. However, live births represent only part of the population in which such adverse outcomes may be detected [28]. Although the proportion of those congenital anomalies that can be attributed to medication exposure is low compared with other environmental causes [29], it is still feasible that some anomalies not captured by our analysis, which considered only live births, could also have been associated with medication use. For example, if the number of cases in the pregnancies lost to ascertainment from our study group A exceeded the number of observed cases, the opposite results to what we reported could have been produced. This is an important limitation, but a problem common to many studies of the associations of birth defects in humans. Under these circumstances the possibility exists for the Yule–Simpson effect (Simpson's paradox) [30], a statistical paradox in which an effect in two groups measured separately (such as congenital anomalies among nonlive births and among live births) is reversed when the groups are combined. This effect has been examined quantitatively [28], illustrated by considering maternal female sex hormone exposure and congenital cardiovascular defects. Clearly, congenital anomalies in nonlive births could have an important impact on OR estimates.

To our knowledge, there is no extensive evidence suggesting that the indication for antilipaemic treatment could itself be a cause of adverse fetal outcome other than a case report of possible intrauterine growth restriction [8]. However, common co-morbidities associated with the use of antilipaemic medications such as hypertension, hypothyroidism and diabetes could increase the risk of adverse fetal outcome. Diabetes, for example, is known to be strongly associated with major congenital anomalies and perinatal mortality [31–33] and is an aetiological factor for abortion [29]. In this study, care was taken to select groups of women that were comparable to account for any possible effects of indication; two comparator groups were selected, with both groups consisting of women who had previously had or currently had an indication for antilipaemic medication treatment.

In this database study we were able to assemble a cohort of all women known to have been prescribed statins or fibrates within a population, and ascertain pregnancy status. This method allowed us to ascertain a denominator (a defined population of exposed individuals) from which the cases (the numerator) are drawn. Controlled epidemiological studies are considered to be more appropriate than uncontrolled case reports in establishing a relationship between gestational drug exposure and pregnancy outcomes in humans [11]. Nevertheless, there is a need for caution in trying to identify patterns, particularly those concerning anomalies that are relatively common, when study populations are small.

Pharmacoepidemiolgical studies based on administrative databases are important and may yield valuable information despite limitations of the incompleteness of information on potential confounders or on certain clinical variables of interest. In this study, we did not have access to information such as the reasons for the termination of a pregnancy. Information on other potential confounding variables such as smoking and folic acid intake were not available. We also acknowledge that an assumption of our study was that those who filled a prescription were also sufficiently exposed to the medicines, but absolute noncompliance with medications obtained by pregnant women is known to be low, at about 8% [34].

An advantage of database studies that include the routine collection of information on dispensed drugs, including name, dose and amount dispensed, is the avoidance of limitations associated with the need for long-term recall by study subjects (that may result in recall bias). They allow the rapid assembly of cohorts that would otherwise be costly and time consuming if done prospectively. Such database research provides valuable information in the investigation of associations that might have an important public health impact.

The prevention of severe hypercholesterolaemia during pregnancy remains desirable. However, for the majority of women discontinuation of antilipaemic drugs such as statins should have little impact on them or their babies. Given the continuing uncertainty over the safety of statins for the fetus, the avoidance of their use during pregnancy remains the best advice; consequently, women on statins should plan their pregnancies to guarantee the best outcome.

In conclusion, we did not detect a pattern in congenital anomalies or find evidence of an increased risk of fetal congenital anomalies in the live-born infants of women filling prescriptions for statins in the first trimester of pregnancy, compared with women on fibrates or those who stopped statins before pregnancy. However, studies of larger populations that include more information on nonlive births are necessary before confirming or refuting an association between statins and fetal congenital anomalies.
